# Choice, deliberation, violence: Mental capacity and criminal responsibility in personality disorder

**DOI:** 10.1016/j.ijlp.2015.04.008

**Published:** 2015

**Authors:** Hanna Pickard

**Affiliations:** Department of Philosophy, University of Birmingham, Edgbaston, Birmingham B15 2TT, UK

**Keywords:** Autonomy, Criminal responsibility, Mental capacity, Personality disorder, Self-harm, Violence

## Abstract

Personality disorder is associated with self-harm and suicide, as well as criminal offending and violence towards others. These behaviours overlap when the means chosen to self-harm or attempt suicide put others at risk. In such circumstances, an individual's mental state *at one and the same time* may be deemed to meet the conditions for criminal responsibility, and to warrant involuntary hospital admission. I explore this tension in how people with personality disorder are treated at the hands of the criminal and civil law respectively in England and Wales: they may be deemed sufficiently mentally well to be punished for their crimes, but not deemed sufficiently mentally well to retain the right to make their own decisions about matters of serious importance to their own lives, including whether or not to continue them. The article divides into four sections. After introducing this tension, Section 2 sketches the nature of personality disorder and the psychology underlying self-directed and other-directed violence. Section 3 addresses the questions of whether people with personality disorder who are violent, whether towards self or others, typically meet the conditions for criminal responsibility and mental capacity respectively, considering in particular whether their underlying desires and values, or their emotional distress, affect their mental capacity to make treatment decisions. Section 4 then considers what we might do to address the tension, within the confines of current legislation. Drawing on *The Review of the Mental Health Act 1983*, I argue that we are ethically justified in involuntarily admitting to hospital people with personality disorder who pose a serious risk to themselves only if we simultaneously undertake to offer genuine help for their future, in the form of appropriate treatment, social support, and better life opportunities — a provision which, as things stand in England and Wales, is sorely lacking.

## 1 Introduction

Consider the following clinical vignette:

P has a long history of involvement with mental health services and is well known to the local police. He has a diagnosis of personality disorder and takes a number of psychiatric medications, including sleeping pills and sedatives. He has had repeated hospital admissions due to overdoses and other forms of severe self-harm, usually in the form of cutting or burning. He drinks regularly, and can become aggressive and threatening, especially when drunk.

The police and the community mental health team are currently trying to locate P, who made an emergency appointment with his doctor this morning. P arrived in a dishevelled, agitated, and emotionally distressed state. Upon questioning, he admitted to intending to kill himself, but ended the appointment abruptly when his doctor suggested a voluntary admission to hospital, saying his mind was made up and no one could help.

P was found late that evening, after spending the day drinking alone in his car, and then returning home and setting fire to his flat. The smoke and flames alerted a neighbour who called for help. P suffered smoke inhalation and minor burns. No one else in the building was injured. P was charged and subsequently convicted of arson that recklessly endangered lives.

Had P been found before setting the fire, he would in all likelihood have been involuntarily admitted to hospital.

P's story is hypothetical, but it will be familiar to many of those who work with personality disorder in mental health and criminal justice services. Personality disorder [PD] is associated with self-harm and suicide, as well as criminal offending and violence towards others. These behaviours overlap only infrequently. Self-harm and suicide has a profound impact on family and friends, but it is rare that the means chosen to self-harm or attempt suicide also put others at direct physical risk. But it does occur. Common examples include attempts to harm or kill oneself by setting fires, like P, or by driving over bridges or overpasses, onto railroad lines, or into oncoming traffic or buildings.

P's story sharply highlights a tension in how people with personality disorder are treated by criminal and civil law respectively in England and Wales. On the one hand, prisons in the UK are populated by people with PD: it is estimated that 64% of male and 50% of female offenders have a personality disorder (NOMS, 2011). Offenders with PD are sometimes diverted from the courts or given a hospital disposal. But as these statistics testify, they are routinely judged criminally responsible and correspondingly held to account.^1^ On the other hand, people with personality disorder who present to mental health services at risk of self-harm or suicide can be admitted to hospital under a Section of the Mental Health Act [MHA] in England and Wales, which allows involuntary detention and compulsory treatment in the presence of a mental disorder (including PD) in cases of risk and irrespective of mental capacity. 2

Good clinical practice aims to avoid involuntary detention and compulsory treatment, especially if previously counter-productive. However, if community management is not a viable option and the risk of harm to self is judged to be serious, the MHA may be used to admit people with a mental disorder to hospital against their will.^3^ Especially with respect to people with a mental disorder where risk of harm to self is stable and long-standing, this may cause clinical disquiet and ethical unease if grounds are lacking for overriding treatment decisions based on mental incapacity under the Mental Capacity Act (MCA), which is the law in England and Wales protecting people who are unable to make treatment decisions for themselves.^4^ Under the MCA, treatment decisions can be made on behalf of patients and in their best interests, when they are unable to do so themselves due to “an impairment of, or disturbance in the functioning of, the mind or brain” which affects their capacity for rational deliberation.^5^ Although the MCA states clearly that every person is presumed to have the mental capacity to make their own treatment decisions and, moreover, that the presence of any condition, such as a mental disorder, cannot in itself justify an assumption to the contrary, it is nonetheless the case that the presence of a mental disorder can affect the ability to rationally deliberate. When this is proven to be so, clinicians can both ethically and legally justify involuntary detention and compulsory treatment of people with mental disorder who pose a risk to self based on mental incapacity under the MCA, potentially quelling any sense of disquiet or unease. However, when mental capacity is retained despite the presence of a mental disorder, then use of the MHA is required instead.

Only people who have a diagnosis of a mental disorder or for whom there are grounds to suggest the presence of a mental disorder in the absence of a previous diagnosis, and so might benefit from assessment, can be involuntarily detained and compulsorily treated under a Section of the MHA due to risk to self.^6^ In England and Wales, people are allowed to self-harm or attempt suicide if there is no diagnosis or grounds suggesting the presence of a mental disorder. Clinical disquiet and ethical unease can result from concern that, in striking contrast to the MCA, the MHA therefore allows discrimination on grounds of mental disorder.^7^ Lingering questions – however inchoate or inarticulate these may be – about retention of mental capacity in such circumstances potentially quell this concern by offering the possibility of non-discriminatory grounds for differential treatment, as all people who lack mental capacity to make their own treatment decisions, mentally disordered or not, fall under the MCA. Especially as mental capacity admits of degrees and borderline cases, it is natural to wonder about the extent to which it is retained during periods of serious risk to self, even if, strictly speaking, the conditions specified by the MCA as determining an ability to make one's own decisions likely obtain. Hence part of the tacit acceptance of use of the MHA in such contexts by practicing clinicians may be an underlying uncertainty about the person's mental capacity — a feeling that something about their state of mind warrants interference if and when they fail to act in what appears to be their own best interests.^8^

P's story sharply highlights the tension in how people with personality disorder are treated at the hands of criminal and civil law because his mental state *at one and the same time* is deemed to meet the conditions required for criminal responsibility, and to warrant involuntary hospital admission. For, again, had he been found before setting the fire, he would in all likelihood have been detained under civil law, as opposed to prosecuted under criminal law. Self-harm, suicide, and violence towards others no doubt demand considered and often robust interventions by the state, and the various purposes of, and potential justifications for, criminal and civil law are of course varied and different. There are no doubt many ways we might attempt to reconcile and rationalize P's treatment by criminal and civil law respectively. But it is nonetheless difficult not to feel, at heart, that P gets a raw deal. For, whichever way he turns, he is subjected to the strong arm of the law — deemed sufficiently mentally well to be punished for his crimes, but not deemed sufficiently mentally well to retain the right to make his own decisions about matters of serious importance to his own life, including whether or not to continue it.

The aim of this article is to explore this tension and make some tentative suggestions about how we might better manage the “awkward questions” that personality disorder raises.^9^ The structure is as follows. Section 2 sketches the nature of personality disorder and aspects of the psychology underlying self-directed violence on the one hand, and other-directed violence on the other. A natural suggestion for resolving the tension is that (cases like P's notwithstanding) the psychology underlying self-directed and other-directed violence associated with PD is fundamentally distinct, with the state of mind associated with harming oneself expressing pathology, and the state of mind associated with harming others expressing a more rational mentality. I argue that this distinction cannot be sustained in a way that supports the difference in treatment by criminal and civil law. Section 3 addresses the vexed questions of whether people with PD who are violent, whether towards self or others, typically meet the conditions for criminal responsibility and mental capacity respectively; and I consider in particular whether their underlying desires and values, or their emotional distress, affect their mental capacity to make treatment decisions. Although all judgements must be made on a case-by-case basis, I suggest that the conditions for both criminal responsibility and mental capacity may often be met. Section 4 considers what we might do to address the tension, within the confines of current legislation. Drawing on *The Review of the Mental Health Act 1983* (Richardson et al., 1999), I argue that we are ethically justified in involuntarily detaining and admitting to hospital those with PD who pose a serious risk to themselves only if we simultaneously undertake to offer genuine help for their future, in the form of appropriate treatment, social support, and better life opportunities — a provision which, as things stand, is sorely lacking.

Before progressing, it is however important to note the difference in the psychological conditions required by law for criminal responsibility as opposed to mental capacity, as prima facie this might be thought to offer a simple resolution to the identified tension. Criminal responsibility depends on the idea of a *voluntary act*. Minimally, the offender must possess cognitive and volitional abilities such that they knew what they were doing at the time of the offence, and had a choice and exercised sufficient control in doing so. Although choice involves a sharp line, in that if a person had a choice at all, they must have had at least two – namely, to act as they did, or to refrain from so acting – the extent to which a person knows or has control over what they are doing permits of degrees and borderline cases, which may serve to mitigate criminal responsibility. But such complexities aside, the basic concept of criminal responsibility depends on a degree of voluntary choice and control — the offender could have not done it.^10^ In contrast, mental capacity as defined by the MCA requires the ability *to rationally deliberate* about a particular matter — where this is understood as a cognitive process involving the abilities to understand, retain, and weigh relevant information, to come to a decision for oneself. This is intended to offer value-neutral conditions for mental capacity, allowing assessments to be made apart from any judgement about the wisdom of the person's decision.

The concepts of choice and of rational deliberation are distinct. Sometimes, of course, the acts of choosing and deliberating are connected: faced with uncertainty as to what to do, we may rationally deliberate in order to make a choice. Nonetheless, it is possible to have a choice – in that one could have not done what one did – even though one did not rationally deliberate prior to choosing. E.g. perhaps one acted without thought, on the spur of the moment, but still, in the moment, one could have stopped. Equally, it is possible to rationally deliberate and come to a clear decision, which one then chooses not to act on. E.g. perhaps one's emotions move one to act in the moment against one's best judgement, prior deliberation notwithstanding. However, despite the differences in the concepts of choice and of rational deliberation, and correspondingly the conditions required for criminal responsibility as opposed to mental capacity, the tension identified nonetheless stands.

The reason is that intuitively, both criminal responsibility and mental capacity are underpinned by considerations of *autonomy*, broadly conceived. It seems correct to hold a person criminally responsible only if what they did was up to them — they could have not done it and so in that sense the action was *their own*.^11^ Equally, when a person rationally deliberates and comes to a treatment decision that counts as *their own* – free from external or internal alien forces, and so it would seem in line with their own desires and values – then their decision ought to be respected, no matter how unwise it may seem to others.^12^ It is beyond the scope of this article to offer a discussion of the many facets of our concept of autonomy and how they might underpin and unify (or fail to) criminal responsibility and mental capacity in law.^13^ The point to emphasise here is that the tension identified cannot be waved aside, simply by pointing to the different concepts and conditions pertaining to criminal responsibility versus mental capacity. For, as soon as we probe deeper, the intuition that something is amiss reappears, now under the guise of the concept of autonomy and what that implies, on the one hand, with respect to our right to insist that a person face the consequences of their (broadly conceived, autonomous) actions, and, on the other, with respect to a person's right to have their (broadly conceived, autonomous) decisions respected. In other words, the difference in concepts and conditions of criminal and mental capacity law notwithstanding, it remains difficult to reconcile the fact that P's mental state at one and the same time can be used to underwrite a finding that he is criminally responsible for endangering others, with, potentially, a refusal to respect his decision to end his own life.

## 2 Personality disorder and violence towards self and others

The ICD-10^14^ describes personality disorder as follows (WHO, 1992):

These types of condition comprise deeply ingrained and enduring behaviour patterns, manifesting themselves as inflexible responses to a broad range of personal and social situations. They represent either extreme or significant deviations from the way the average individual in a given culture perceives, thinks, feels, and particularly relates to others. Such behaviour patterns tend to be stable and to encompass multiple domains of behaviour and psychological functioning. They are frequently, but not always, associated with various degrees of subjective distress and problems in social functioning and performance.

It also stipulates the following criteria as diagnostic guidelines:

(a) markedly disharmonious attitudes and behaviour, involving usually several areas of functioning, e.g. affectivity, arousal, impulse control, ways of perceiving and thinking, and style of relating to others;
(b) the abnormal behaviour pattern is enduring, of long standing, and not limited to episodes of mental illness;
(c) the abnormal behaviour pattern is pervasive and clearly maladaptive to a broad range of personal and social situations;
(d) the above manifestations always appear during childhood or adolescence and continue into adulthood;
(e) the disorder leads to considerable personal distress but this may only become apparent late in its course;
(f) the disorder is usually, but not invariably, associated with significant problems in occupational and social performance.

Unlike e.g. schizophrenia and mood disorders, PD is not conventionally understood as an illness or disease; nor is it typically characterised by delusions or psychotic thinking. Rather, as the name implies, it is a disorder of *the personality*: an enduring tendency of mind and pattern of behaviour. Metaphorically speaking, it is a condition that is internal to the kind of person someone is, a part of who they are.

Very roughly, personality is the set of standing, psychological traits that comprise the ways a person is inclined to think, feel, and act. Personality disorder occurs when this set of traits is maladaptive, causing personal distress and impairment in social, occupational, or other important contexts: the ways a person is inclined to think, feel, and act do them harm, directly or via effects on functioning. Diagnosis with a PD requires that these traits are stable, pervasive, difficult to control or change, and markedly different from cultural expectations. Equally, the extent to which they are maladaptive must be sufficient to warrant clinical attention. Nonetheless, all of us possess traits that sometimes incline us to think, feel, and act in ways that cause us harm. Personality disorder lies on a continuum with normal human personality.

The broad category of ICD-10 personality disorder is divided into a variety of types. These include Paranoid, Schizoid, Dissocial, Emotionally Unstable (there is both an Impulsive and Borderline sub-type), Histrionic, Anankastic, Dependent, Anxious (also known as Avoidant), and Mixed and various other types.^15^ A person cannot be diagnosed with any particular type of PD unless the general diagnostic guidelines (a)–(f) are met. But, given this, what kind of PD a person has will depend on what kind of personality they have: on the nature of the maladaptive tendency of mind and pattern of behaviour.

With respect to aetiology, PD is associated with genetic factors (Jang & Vernon, 2001) but also environmental conditions (Paris, 2001). These include dysfunctional families, where there is breakdown, death, institutional care, and parental psychopathology; traumatic childhood experiences, with high levels of sexual, emotional, and physical abuse or neglect; and social stressors, such as war, poverty, and migration. There are high levels of co-morbidity among personality disorders, and between personality disorders and psychotic disorders, eating disorders, anxiety, depression (Hayward & Moran, 2008; Lenzenweger, Lane, Loranger, & Kessler, 2007), and especially substance abuse (Lenzenweger et al., 2007; Thomas, Melchert, & Banken, 1999). Finally, a study using DSM personality disorder classifications found that people with Cluster B PDs,^16^ which corresponds roughly with a combination of ICD-10 Dissocial, Emotionally Unstable, and Histrionic personality disorders, are 10 times more likely to have a criminal conviction and 8 times more likely to have spent time in prison compared to those without (Coid et al., 2006); equally, there is also a strong association between these PDs and self-harm, suicide and violence towards others (Coid et al., 2006); with self-harm further associated with childhood sexual abuse, and violence towards others further associated with childhood psychical abuse (Waxman, Fenton, Skodol, Grant, & Hasin, 2014).

As this picture suggests, people with personality disorder often come from backgrounds of extreme psycho-social and economic adversity, have been victims of trauma, neglect, and abuse in childhood, and lead chaotic, fragmented, and despairing lives as adults, suffering high levels of psychological distress and social marginalization. They also place a heavy burden on psychiatric, medical, social, legal and forensic services. Within psychiatry, people with personality disorder have long been stigmatized as the patients “no one likes” and personality disorder itself considered impossible to treat. But, over the past two decades, this has started to change, with emerging evidence-based consensus that improvement and even recovery is often possible, through a range of psychological treatments, sometimes offered in conjunction with (typically time-limited) medication (NICE, 2009; NIMHE, 2003; Wood, Bolton, Lovell, & Morgan, 2014). These psychological treatments work by helping people alter or at least better manage the more problematic traits comprising their personality. Although all psychological treatments are multi-pronged and complex, and there are of course differences between them, to be effective they must to some extent target criterion (a) of the ICD-10 diagnostic guidelines, addressing the maladaptive attitudes and behaviour, including affectivity, arousal, impulse control, ways of perceiving and thinking, and style of relating to others.^17^ The basic assumption underlying psychological treatment is that personality is not fixed, but at least to some extent flexible. Personality traits develop in response not only to genetic but also environmental factors: ways of thinking, feeling, and acting are learned, and can therefore to some extent be unlearned, altered, and adapted through treatment. In other words, people with personality disorder can change in their cognitive, affective, behavioural, and interpersonal traits, and learn to do things differently.

Self-harm, suicide, and violence towards others of course need not be associated with mental disorder of any sort, and can have many different underlying motivations.^18^ But in the context of personality disorder and its associated background, they are clinically understood to have a set of specific *functions*.

Consider first violence towards others. From our shared cultural perspective, its functions are not difficult to fathom. But in addition to the use of violence as a direct means to procuring material goods, violence can serve (but is not restricted to) the following ends^19^:

(1) *Affect regulation*. The expression, release, and communication of aggressive impulses and strong emotions, especially anger.
(2) *Social and/or interpersonal dominance and/or control* and the various benefits it accrues, often in conformity with cultural stereotypes of masculinity.
(3) *Revenge*. Retaliation/retribution towards those who have perpetrated psychological and/or physical harm to the agent or someone they care about.
(4) *Protection from future harm*. Violence can signal a willingness to retaliate/seek retribution and so act as a deterrent, when directed towards those who have perpetrated past harm or are threatening to perpetrate future harm.

When associated with PD, violence towards others may fulfil such functions. First and foremost, it can be driven by aggression and anger — a way of expressing, releasing, and communicating strong emotions. This may have roots in childhood experiences of violence in the family or wider community, where there may have been little opportunity for alternative ways of managing anger and other emotions to be learned. Indeed, violence can be part of the fabric of a community, accepted and even expected, as necessary to success and survival. Especially given such a background, violence may also serve for those with PD as a means to establish control when they perceive a threat to their physical or psychological wellbeing, including the potential for shame that is carried by past or ongoing victimhood and mistreatment. And, as with all patterns of behaviour that fulfil various functions effectively, violence can become *a habit*. Interventions for violence associated with PD typically involve creating a narrative understanding of how past experience has impacted on development; identifying and avoiding triggers; reflecting on emotions, and learning alternative ways of managing aggression, anger, and resolving conflict — all within a therapeutic environment that offers support, compassion, and understanding.^20^ But the basic point is that from our shared cultural perspective, personality disordered violence towards others appears *rational*: a natural way of expressing, releasing, and communicating aggression, anger, and other emotions, and a means to achieving ends we can easily understand why people in a variety of contexts would have.

In contrast, the functions of self-directed violence are less easy to fathom from our shared cultural perspective. Indeed, it can be difficult even to conceptualise self-harm and suicide as *violence* at all.^21^

Violence is standardly defined as behaviour involving physical force intended to hurt, damage, or kill. There is no stipulation that the victim and perpetrator cannot be identical. Not all forms of self-harm or suicide involve physical force as we commonly understand it. E.g. over-dosing or self-poisoning may aim at damage or death, but not employ physical force. Equally, although self-harm is typically deliberate, it can be unconsciously motivated: self-inflicted injury may sometimes pass as an “accident” even to the injurer. But such caveats notwithstanding, many central instances of self-harm and suicide – like cutting, scratching, burning, or smashing one's body parts with weapons or against walls; swallowing blades or sharp glass or inserting them under the skin or in orifices; hanging oneself, shooting oneself, or throwing one's body under trains or off buildings – straightforwardly meet the standard definition of violence.

From our shared cultural perspective, such behaviour can seem the epitome of *irrationality*. For what could possibly be gained by doing it? People who do not self-harm themselves, or lack personal or professional experience with those who do, may have limited resources for understanding what ends it could serve and hence why anyone would act thus. We commonly think that, when people can act so as to avoid harm to themselves, they do. But people who self-harm do precisely the opposite of this — they act to directly harm themselves. We may therefore find ourselves inclining to the view that self-harm can only be understood as an expression of underlying pathology. For, without an understanding of its functions, no sense can be made of why any person would do this *if they could help it*.

It is important to emphasise again that self-harm and suicide need not be associated with mental disorder of any sort, and that there can be many different underlying motivations. But in a context of personality disorder and its associated background, clinical understanding and patient self-reports reveal self-harm as serving at least six ends that help explain why, in general, people with personality disorder may do so.^22^

Self-directed ends of self-harm:

(1) *Affect regulation*. People with PD suffer great emotional distress. Self-harm can be a way of managing strong emotions, perhaps especially anger and shame, which can be particularly strong in those who come from backgrounds of psycho-social and economic adversity and suffered childhood neglect and abuse. It may offer relief in various ways: by distracting from emotional pain by replacing it with physical pain and/or releasing endorphins; by providing a way of expressing, releasing, and acting on anger and aggression, just as with violence towards others; or, in contrast, by allowing people to *feel something* in the face of dissociation and emotional numbing. In other words, self-harm can offer short-term relief from negative emotional experience: it is a coping mechanism.
(2) *Self-punishment*. People with PD typically have extremely low self-esteem, and often believe they are bad and deserve to be harmed. Self-harm can be both expressive of and explained by this, especially in face of strong emotions of anger and shame.

Other-directed ends of self-harm:

(3) *Communication*. Self-harm can be a way of communicating internal distress by symbolizing emotions in concrete, physical form: “the public expression of […] private pain” (Adshead, 1997, p. 11). People with personality disorder often struggle to identify and talk about their feelings. Self-harm offers a powerful way of demonstrating what they are going through, and, potentially, thereby seeking care and help.
(4) *Other-punishment*. It is common to feel aggressive, angry, and want to be violent towards those who have harmed us or others we care about. Self-harm can offer a safe way of expressing anger and related emotions, when violence towards others is deemed unacceptable. This function of self-harm correlates with the experience of being attacked that self-harm can provoke in others, as it becomes like a symbolic weapon, turning anger towards others inwards on the self, while yet communicating it.

Self-directed and other-directed ends of self-harm:

(5) *Control*. Self-harm can create a sense of empowerment and control by establishing ownership over one's body in face of the experience of being helpless and violated. This may be especially important for those with childhood experience of physical and sexual abuse. For people whose bodies have been harmed by others, it can be an act both of reclaiming their body and, to use Anna Freud's (1992) concept of identification with the perpetrator, establishing that it is they, and no one else, who now has dominance and control.

A desire for death:

(6) *The continuum with suicide*. When associated with personality disorder, the desire to kill oneself is typically understood as expressive of hopelessness and despair — a desire for permanent escape from the suffering of living. In contrast, self-harm offers short-term relief, and hence, as Anna Motz has eloquently argued, can be seen instead as an act of hope (Motz, 2009b) — an affirmation of life. However, although the idea of suicide is distinguished from self-harm by nature of intent – to die rather than to do harm or damage – in reality the distinction between them is often unclear, with people unsure and, even more, indifferent with respect to intended outcome as well as reckless in methods, so that self-harm regularly and knowingly risks death, even if it does not clearly and consciously aim at it.^23^

The extreme anguish, anger, and shame that can underlie self-harm are typically linked to the adverse and traumatic backgrounds typical of those with PD. In this context, self-harm is a means to ends that we can understand why people would have, such as relief from negative emotional experience, expression and communication, seeking care, attacking or punishing those perceived to have done wrong (including oneself), and gaining a sense of power and control in the face of feelings of helplessness and violation. In so far as it fulfils these functions effectively, self-harm can become *a habit*. Interventions for self-harm associated with PD typically involve creating a narrative understanding of how past experience has impacted on development; identifying and avoiding triggers of self-harm; improving self-esteem; reflecting on emotions, and learning alternative ways of managing the anguish, anger, and shame — all within a therapeutic environment that offers support, compassion, and understanding.^24^ But the basic point is that, via proper attention to clinical understanding and self-reports, self-harm is like violence towards others in that it can be understood as *rational*: a way of expressing, releasing, and communicating anguish, anger, shame, and associated beliefs and impulses, and so providing relief from these as well as achieving a raft of other ends that it is understandable to have — especially given the life circumstances and genuinely available alternative options and resources, both internal and external, associated with PD.

P's story is unusual: it is rare for people with personality disorder to put others at direct physical risk when they deliberately try to harm or kill themselves. But it is possible to understand why it happens. Self-directed violence may be a way of turning anger towards others inwards onto the self, as well as a means of communicating it, and, potentially, symbolically or actually punishing others. In the grip of strong emotions, anger towards others that is being directed onto the self may end up being turned back onto others, via recklessness, indifference, and, potentially, conscious or unconscious motivations, with respect to the means chosen to self-harm or attempt suicide. In other words, P's state of mind at one and the same time may contain aggressive impulses directed both towards himself and towards others.

Summing up, people with PD use both self-directed and other-directed violence for various and sometimes overlapping purposes, including but not restricted to affect regulation, expression and communication of emotions, and the establishment of power and control. There is of course an important difference: self-directed violence does not indicate a willingness to be violent *towards others*, and so may mark a difference in attitude towards morality and law, as well as ipso facto in criminality. But it is not possible to draw a sharp line between self-directed violence as *pathological*, and other-directed violence as *rational*. They both display instrumental reasoning, and serve ends that are evidently desirable or valuable. Hence what distinction there is between them cannot comfortably support the difference in treatment by criminal and civil law.

## 3 Emotions, deliberation, choice

Most of our actions do not flow from a process of rational deliberation — they just flow. Consider, for instance, an ordinary morning routine: the alarm clock rings and we get up and get washed, dressed, ready for work and the school run. These actions are guided by reasons and serve various ends – to get to work, to get the kids to school – without any need for us to reflect on what we are doing and decide which action out of the range of available alternatives we ought to perform. We just perform the morning routine. Of course, within this routine, we may at times be stopped short, and required to deliberate and decide what to do. Finding there's no cereal left in the box for breakfast, we face the question what to eat instead? But action does not depend on such deliberation in order to be guided by reasons and subject to choice and a degree of control — at least in the minimal sense identified above as a condition of criminal responsibility. In full flow of acting, we yet have the capacity to choose not to act — at the very least, to stop the flow.^25^

Violence, especially if habitual, can be part of the flow. There may be no antecedent process of deliberation, and yet, as described in Section 2, it can be guided by reasons and serve various ends. In addition, we are inclined to believe that violence is on many occasions subject to choice and a degree of control — precisely in that the person could have not done it. In general, people are capable of choosing not to act violently — to stop the flow.

The evidence for this presumption is straightforward: when sufficiently motivated to refrain from (self-directed or other-directed) violence in a variety of contexts, people do. Consider, for instance, a man who “sees red” and routinely gets into conflicts and resorts to violence — except when in view of a policeman. On such occasions, he is highly motivated not to lash out, which he would otherwise do, lest he be detained and charged with common assault. This is the classic “policeman at the shoulder” test. Our natural understanding of this test is that it shows that this man has the capacity to choose not to be violent and control his aggression — a capacity, of course, that he only exercises when sufficiently motivated to do so. Similar kinds of “at the shoulder” tests exist for self-directed violence, as when e.g. a person who routinely self-harms ensures that their children never witness it, or stops “cold turkey” as a condition of participating in a therapy group.^26^ There is a basic, commonsense distinction between what a person can do but won't (because they don't want to) as opposed to what a person wants to do but can't (because they lack the capacity). In so far as violent behaviour (in those with or without PD) is responsive to incentives, it appears to be subject to choice and a degree of control. Exercising this capacity and desisting from violence would seem to be, in general, something people can do but sometimes don't, as opposed to something they want to do but can't.^27^

Nonetheless, it is extremely important to recognize how difficult it may be for people to exercise the capacity to desist from violence, especially when, as with personality disorder, it may both be habitual and serve valuable ends, which people may lack genuine or perceived alternative means of achieving. E.g. in so far as violence offers affect regulation, refraining from violence may require a person to undergo emotional distress and bear feelings of extreme anger and shame, unless and until alternative ways of coping with these are learned. They may, in other words, be subject to a form of internal duress if they choose to refrain. Equally, the general presumption that violence is subject to choice and a degree of control may, of course, be defeated in particular circumstances. Perhaps sometimes a person who “sees red” becomes so angry that something “boils over” or “snaps” and choice and control is lost — they are not then able to stop the flow towards violence.^28^ But in general, the presumption that people with PD meet the conditions for criminal responsibility with respect to violent crimes seems plausible, even if on particular occasions this is mitigated by internal duress or loss of control.

What now of mental capacity? Recall that the MCA understands mental capacity as a cognitive process involving the abilities to understand, retain, and weigh relevant information, to come to a decision for oneself. There are two standard concerns that might be thought to bear on whether people with PD possess mental capacity when risking serious harm to self. The first pertains to the nature of the desires and values they use in the cognitive process of deliberating. The second pertains to their underlying state of emotional distress. Let us take these in turn.

Weighing information requires a background set of desires or values for it to be weighed against. Although not encoded in the MCA, there is broad agreement within the relevant literature on mental capacity and autonomy that, following Brock and Buchanan, these desires and values must be stable and enduring commitments.^29^ When people make decisions with serious consequences that appear to be unwise or against their best interests, these should not be based on whim. Tomorrow's regret offers a reason to override today's decision, as it provides an indication that the desires and values shaping the decision are not part of a person's authentic self or standing goals, but due to sudden impulse. A degree of diachronic continuity is therefore an important part of identifying the desires and values that shape autonomous decisions.^30^ Signs of ambivalence and actions that are out of character offer evidence that this condition is not met.

Returning to P, as his story is told, there are signs of ambivalence. Despite claiming his mind was made up and no one could help, P made an emergency appointment with this doctor, in effect asking for help. Such ambivalence is important when considering self-harm and suicide in relation to PD. If a person is whole-heartedly committed to a course of action, they will usually try to avoid contact with people or services that might stand in their way. When people with PD voluntarily contact services that may prevent them from harming or killing themselves, there is reason to think they are not committed to doing so and hence to override treatment decisions.

However, people with PD may also try to avoid contact, or to indicate in advance that, if it does occur, they do not wish to be treated. This was so in Kerry Wooltorton's case, who was allowed to die from self-poisoning, having written a letter stating clearly that she did not wish to receive life-saving but only palliative treatment.^31^ In some cases, there may be no signs of ambivalence or reason for thinking that the desires and values underlying the decision do not express stable and enduring commitments. Although people with PD (especially Emotionally Unstable PD) may be impulsive and reckless, with an unstable sense of self, the desire to self-harm may be relatively constant and pervasive. Indeed, people sometimes have a clear and strong commitment to killing themselves if life becomes unbearable, as this can offer control and solace — they know they have an escape from pain and suffering if and when they need it.^32^

In this respect, it matters that personality disorder is not understood as an illness or disease, but as internal to the kind of person someone is. For there is no “authentic” or “non-personality-disordered” self who possesses a stable sense of self-worth and is concerned for their future, to which we can appeal in order to explain why the self-destructive desires and values under consideration are not the person's own. Rather, as described in Section 2 above, people with personality disorder may devalue themselves tremendously, and have little commitment to life or wellbeing. Although there may be some cases where there is evidence that the desires and values underlying a decision are not stable and enduring commitments, in other cases this consideration cannot be adduced to override treatment decisions.

Consider next the second standard concern bearing on mental capacity, namely, the fact that people with PD who want to self-harm or kill themselves are typically in great emotional distress. Within clinical contexts, it is usually presumed that people with PD will pass a standard mental capacity assessment, even when emotionally distressed. This may be one reason why tools such as the Mac-CAT-T are rarely used: a recent preliminary study conducted by George Szmukler that administered the Mac-CAT-T to two personality disordered patients who refused treatment for self-harm found them to have capacity (Szmukler, 2009).^33^ But despite this presumption, there may yet be concern that emotional distress nonetheless disrupts the ability to make autonomous decisions, and uncertainty as to whether the available tools accurately assess this.

This concern may arise in part because of the culturally prevalent conception of emotions as disturbances to reasoning and rationality. Indeed, in one of the rare cases where the MCA in addition to the MHA was considered by the courts as a basis for overriding treatment refusal, a personality disordered offender who attempted to self-harm by refusing food was determined to lack mental capacity, on grounds that his ability to weigh information “was impaired by *the emotions* and perceptions he had at the time … related to his personality disorder … His spectacles are *blinkered* … Although he weighs facts, his set of scales are not calibrated properly …” (R v Collins and Ashworth Hospital ex parte Brady, at para 59; my italics).^34^ Jodi Halpern has offered a similar argument with respect to the (non-personality disordered) case of Ms G, who refused post-operative care and subsequently died when her husband left her for another woman immediately after she had both her legs amputated. Halpern argues that Ms G's decision should have been overridden because the emotional trauma of these circumstances caused her to be unresponsive to evidence and unable to imaginatively think through future possibilities, including the possibility of finding future happiness without her husband.^35^

Emotions can indeed sometimes “blinker” our ability to deliberate. E.g. if we are so aroused that we find it difficult to retain information, or if our attention remains focused on certain features of our situation to the exclusion of others, as it arguably did with Ms G, who may well have been in a state of shock. On the other hand, there is increasing evidence that, in general, emotions are not disturbances to reasoning and rationality, but essential to the good working of all deliberative processes.^36^ Our emotions can guide us in making correct decisions before we are able to articulate our reasons or know why we are deciding as we are.^37^ Equally, our emotions can guide us away from our purportedly all-things-considered best judgments, showing us what we really ought to do when, as it happens, our deliberative processes have failed to be true to our authentic selves.^38^ Within the literature on clinical ethics, it is increasingly acknowledged that emotions – including those which are extremely strong and distressing – can be responsive to and illuminating of real features of our situation that matter to us deeply, and so may help us come to better understand ourselves and decide what to do.^39^ If the emotional distress of those with PD who want to self-harm or commit suicide is adduced as sufficient grounds for overriding treatment decisions, whether this argument is offered in principle or alternatively in accordance with the law as currently encoded by the MCA, that can only be because something particular about their emotional state impairs the capacity for autonomous rational deliberation. What could this be?

In discussing Ms G, Robin Mackenzie and John Watts write: “The tragedy is that in this case therapy or treatment [e.g. for emotional trauma] might have corrected her assumptions regarding future quality of life, and a decision to accept the lifesaving treatment might have resulted” (Mackenzie and Watts, 2011, p. 33). The suggestion is that Ms G's emotionally traumatised state rendered her incapable of seeing any possibility of future happiness, while had she not refused treatment, but instead continued to live and received therapy, she would have been able to make a more accurate assessment. Transposing this suggestion to the present context suggests that the great emotional distress experienced by people with PD who self-harm or attempt suicide renders them incapable of seeing any possibility of future happiness — *when a more accurate assessment would admit hope and that treatment for their personality disorder could help them to see this*. In other words, the grounds for overriding treatment refusal would depend on their emotional state blinding them to the genuine possibility of a better life. Arguably, from the perspective of the law as currently encoded by the MCA, this could call into question their ability to understand the information relevant to their decision in an adequate manner.

However, there are two grave difficulties with this suggestion. First, there may realistically be very little hope of a genuine possibility of future happiness. Personality disorder is enduring and pervasive, and people may have been living with great emotional distress, pain, and suffering for years if not indeed decades. The inductive evidence may be overwhelmingly in favour of the belief that there is little hope for improvement let alone recovery, and every reason for despair.^40^ Second, such possibility as there is likely depends on the provision of effective treatment, no doubt alongside social support, and educational, and occupational opportunities. Although, as described in Section 2 there is increasing evidence that personality disorder can be treated through a range of psychological interventions, there is a striking lack of provision of services. Many people with personality disorder simply do not have access to specialist treatment, adequate social support, or meaningful training and work opportunities.

Judgements of mental capacity and criminal responsibility must of course be made on a case-by-case basis. Ambivalence, unstable desires and values, and emotions which blind people to genuine future possibilities, can provide grounds for questioning mental capacity and potentially overriding treatment decisions under current law. Equally, emotions can impact on the capacity for choice and control, mitigating criminal responsibility. But, in many cases, people with PD who engage in self-directed violence are likely to possess mental capacity, and those who engage in other-directed violence are likely to meet the conditions for criminal responsibility. There is therefore no avoiding the impression that people with personality disorder, like P, can get a raw deal: deemed sufficiently mentally well to be punished under criminal law, but not sufficiently mentally well to retain the right to make their own treatment decisions during period of high risk, despite the fact that they possess mental capacity to do so. Their autonomy is respected by one branch of the law while disregarded by another. Aside from the possibility of significant legal reform in either branch of law, what can we do to address this situation within the confines of current legislation?

## 4 Addressing the ethical dilemma: pragmatism, beneficence, and autonomy

These “awkward questions” for the law have arisen not only because of the complexity of personality disorder, but because of our response to this complexity. On the one hand, we do not want to stand by and idly watch as people self-harm and die. Many of us despair that members of our society suffer the high levels of psychological distress and social marginalization typical of PD. This sentiment may be compounded when we recognize the contribution of underprivileged backgrounds of extreme psycho-social and economic adversity, often containing childhood trauma, abuse and neglect. It may also be particularly strong in clinical staff, who have chosen a profession guided by a duty of care: when the MHA is used to involuntarily admit people with PD to hospital at risk of self-harm or suicide, it may well be done out of genuine benevolence and concern. But, on the other hand, as a society we have not succeeded in addressing social and economic inequality and childhood mistreatment, or provided adequate specialist treatment, social support, and educational and occupational opportunities for those with PD.

*The Review of the Mental Health Act 1983* asserts: “The Committee is convinced that if society is to impose a duty to comply with care and treatment on some of those who suffer from mental disorder it must impose a parallel duty on health and social care authorities to provide an appropriate standard of care and treatment for those subject to compulsion” (Richardson et al., 1999, p. 1). The *NICE Guidelines for Self-harm: the short-term physical and psychological management and secondary prevention of self-harm in primary and secondary care* are explicit that the standard and experience of care is frequently unacceptable (NICE, 2004). People with PD have a high frequency of hospital admission: they may be (voluntarily or involuntarily) admitted to hospital in periods of high risk, but then discharged when risk is lower but without any change in personality or plan for treatment. Moreover, when the admission is involuntary, they may understandably resent the intrusion and reject what limited help exists, so that relationships with clinical staff are worsened, potentially even evoking early life experiences of coercion and mistreatment. The use of the MHA to involuntarily detain people with PD who possess mental capacity in order to protect them from self-directed violence may be motivated by benevolence and concern, but it is arguably not only discriminatory in failing to respect the autonomy of those with mental disorder but in other ways unethical: relieving our collective conscience of the burden of self-harm and suicide, but doing little to genuinely help those who are violent towards themselves.

The solution to this ethical dilemma is in principle, if not in practice, straightforward: improve the provision of specialist treatment, social support, and life opportunities for those with PD, as mandated by *The Review of the Mental Health Act 1983*.

This would have two related effects. First, it would allow involuntary detention under a Section of the MHA not only to serve the short-term end of averting self-directed violence, but also the long-term end of providing treatment which offered the possibility of genuine improvement and recovery. The harm of failing to respect autonomy, which such uses of the MHA arguably perpetrate, could then be counter-balanced by the potential lasting good of this outcome.

Second, it could potentially offer grounds for justifiably overriding treatment decisions under the MCA, based on concerns about mental incapacity. The difficulty transposing the insights from Ms G's case to people with PD is due to the fact that there may be little hope for improvement let alone recovery, in absence of the provision of effective treatment, social support, and better life opportunities. If we undertake to provide these as a condition of using the MHA to involuntary detain those with personality disorder at risk of harm to self, and communicate this during the process of doing so, we transform the future possibilities that exist. A person who, during this process, was blinded to these possibilities due to emotional distress and did not weigh them in the decision to refuse hospital admission and treatment would therefore be unresponsive to (newly available) evidence and unable to imaginatively think through (newly available) future possibilities. Grounds for overriding treatment refusal under the MCA might therefore exist, and help to ethically justify involuntary hospital admission under a Section of the MHA — given, that is, that we were indeed committed to genuinely providing these resources in the particular case at hand, and communicated this effectively to the person.^41^

Although the MHA grants the power to involuntarily detain and compulsorily treat, psychological treatment for PD requires co-operation to be effective. People with PD must work to change or better manage problematic personality traits, which requires willing participation, motivation and resolve, as well as positive relationships with staff. Clinical skill in framing choices, respecting autonomy, and establishing trust in order to support decision-making, is important when working to engage people with PD whose risk is high. For hospital admission and specialist treatment is much more likely to be accepted and effective if presented as an offer rather than a threat. Hence, although the ethical dilemma described above can be addressed by the provision of specialist treatment, social support, and life opportunities together with the sincere and clinically skilled *offer* of these during the process of detaining someone under a Section, there may be no clinical and correspondingly ethical value in compulsorily treating people with PD if they continue to refuse treatment post-admission, as outcomes are likely to be poor. Indeed, compelling treatment in this context could be seen to compound the failure to respect autonomy, rather than serving to counter-balance it with the possibility of a positive outcome.^42^

The moral of P's story is this: it can be vexingly difficult to establish, in any particular case, whether a person with PD has mental capacity or meets the conditions for criminal responsibility. When the violence is self-directed, we may find ourselves inclined to intervene benevolently but also paternalistically, even when our best guess is that mental capacity is retained, and seek an involuntarily admission to hospital under a Section of the MHA despite the failure to respect autonomy this may perpetrate. When the violence is other-directed, we may find ourselves inclined to punish, even when our best guess is that the underlying state of mind may, at least in part, make the action less than fully autonomous and hence mitigate criminal responsibility. It may be unrealistic to think these responses or either branch of the law are likely to change. But we can begin to address the tension and circumvent at least some of the ethical problems raised, even in the face of uncertainty as to the person's underlying state of mind, by doing what a principle of beneficence recommends in any case: when we intervene in the life of a person who suffers from personality disorder to limit self-directed violence, or to punish other-directed violence,^43^ we simultaneously commit to providing the necessary resources to make their life better.

## Footnotes

1 Personality disorder does not usually fall under The M'Naughten Rules: “to establish a defence on the ground of insanity, it must be clearly proved that, at the time of the committing of the act, the party accused was labouring under such a defect of reason, from disease of the mind, as not to know the nature and quality of the act he was doing; or, if he did know it, that he did not know he was doing what was wrong” (*Queen v. M'Naghten* (1843) 10 Cl and Fin 20). Nor is a plea of diminished responsibility due to mental abnormality under Section 2(1) of the Homicide Act 1957 necessarily available to reduce a murder conviction to manslaughter.

2 Statistics documenting use of the MHA in cases of personality disorder are not available, as data pertaining to category of mental disorder has not been collected since 2008.

3 Cf. *The NICE Guidelines on Self-harm: the short-term physical and psychological management and secondary prevention of self-harm in primary and secondary care*: “.... although it is not a common occurrence, compulsory treatment [under the MHA] can include medical and surgical treatment for the physical effects of self-harm if the self-harm can be categorised as either the consequence of or a symptom of the patient's mental disorder” (NICE, 2004, p. 92). Note that, although good clinical practice will not use the MHA unless risk to self is judged to be serious, the MHA itself does predicate the power to involuntarily detain and compulsorily treat on a requirement of *serious* risk to self or others, but only on the need for patient “health and safety” or “the protection of other persons” (Sections 2 and 3); there is a severity requirement only with respect to restriction orders imposed due to risk of harm to others (Section 41).

4 The MCA has very occasionally been deployed to remove the right to refuse treatment for self-inflicted injury with people with personality disorder. For discussion see Peay (2011a, 2011b).

5 See Sections 1–4 of the MCA. The nature of mental capacity and its connect to rational deliberation are discussed further below.

6 Or who present to the police in need of removal from a public place to a “place of safety”, such as a police station or a hospital, where they can then be kept for 72 h and assessed by an approved mental health professional or clinician. See Section 136 of the MHA.

7 For discussion see Hope (2004) and Richardson (2013). This feature of the MHA contravenes one of the core guiding principles of *The Review of the Mental Health Act 1983:* “The desire to promote the principle of non-discrimination on grounds of mental ill health has been fundamental to the Committee's approach, and this has led to an emphasis on patient autonomy” (Richardson et al., 1999, p. 3). See too Sections 2.14–2.16.

8 A further reason may be fear of investigation and litigation if patients with personality disorder commit suicide or seriously harm themselves when in care.

9 To borrow a phrase from Peay (2011b).

10 Note that this modal claim can be interpreted in various ways; one influential view is that the offender must have had a *fair opportunity* not to have done it (Hart, 2008). See Ashworth (2009) ch. 6 for legal discussion. For philosophical discussion of its interpretation in relation to the traditional problem of free will, see Holton (2010), List (2014), and Whittle (2014).

11 Cf. Ashworth: “To proceed to conviction without proof of voluntary conduct would be to fail, in the most fundamental way, to show respect for individuals as rational, choosing beings. More generally, if people were liable to conviction despite doing nothing, or because something had been done *to* them, this would fail to respect their autonomy” (2009, p. 86). See too Ashworth (2009 ch. 2). This idea is also important within contemporary just deserts theory. For discussion, see e.g. von Hirsch (1976, 1993), von Hirsch and Ashworth (1998), and McDermott (2001).

12 This is one of the basic principles of medical ethics. See Beauchamp and Childress (2009). For discussion with respect to the MCA, see e.g. Mackenzie and Rogers (2013) and Richardson (2013). It is also endorsed by *The Review of the Mental Health Act 1983* which emphasises the importance of patient autonomy understood as “the freedom to decide for oneself, the ability to make choices which others will respect” (Richardson et al., 1999, p. 18).

13 For a survey of the different facets of our concept of autonomy, see Stoljar (2006). Radoilska (2012) is a collection on autonomy in relation to mental disorder.

14 The ICD-10 is broadly similar to the DSM-IV-TR and DSM-5, which define personality disorder as an enduring pattern of experience and behaviour that deviates markedly from the expectations of an individual's culture and is manifest in (at least two of the following areas of) cognition, affectivity, interpersonal functioning, and impulse control. The pattern must be inflexible and pervasive across a broad range of personal and social situations; lead to clinically significant distress or impairment in social, occupational, or other important areas of functioning; be stable and of long duration, with its onset traceable at least to adolescence or early adulthood; and not be better accounted for by diagnosis with another mental disorder or due to direct physiological effects of a substance (e.g. a drug of abuse) or a general medical condition (e.g. head trauma) (APA, 1994, 2013).

15 These correspond roughly but not exactly to the types listed in DSM-IV and DSM-5. The DSM divides the broad category of personality disorder into three sub-categories or “clusters”. Cluster A comprises paranoid, schizoid, and schizotypal personality disorder. These are more colloquially known as the odd and eccentric or “mad” PDs. Cluster B comprises narcissistic, borderline, histrionic, and anti-social personality disorder. These are more colloquially known as the dramatic, emotional, and erratic or “bad” PDs. Cluster C comprises obsessive-compulsive, avoidant, and dependent personality disorder. These are more colloquially known as the anxious and fearful or “sad” PDs.

16 See fn 15.

17 For further discussion of some of the philosophical and psychological elements within the therapeutic process, see Pearce and Pickard (2013), Pickard (2011, 2013, 2014, 2015), and Wood et al. (2014). For two classic discussions of some of the clinical challenges of working with people with personality disorder, see Main (1957) and Vaillant (1992).

18 To take but a few examples, self-mutilation and the endurance of pain is a part of many religious and cultural rituals; suicide may be a rational, considered, and dignified choice for those who are terminally ill, as well as an act of political protest or moral or religious faith; combatant violence is considered normal and justifiable within war. For discussion see Favazza (1987) and Fincham, Langer, Scourfield, and Shiner (2011).

19 It is important to distinguish what has been labelled “reactive” or emotionally “hot” violence associated with personality disorder, from what has been labelled “instrumental” or emotionally “cold and calculating” violence associated with psychopathy. For discussion of this distinction and the empirical data supporting it, see e.g. Blair, Mitchell, and Blair (2005), Howells (2009), and Raine (2013). The focus of this article is “reactive” or “hot” violence associated with personality disorder. However, the labelling is unfortunate, as reactive violence is also instrumental, in the sense of serving various functions or ends, as specified above.

20 For general discussion of violence and personality disorder, see McMurran and Howard (2009). McMurran (2009) offers a short summary of *Stop&Think!* — an intervention designed to promote pro-social problem solving as an alternative to violence. For discussion of the role of narrative in effective therapy for personality disorder, see Pickard (2014).

21 For discussion see Motz (2008) and Pickard (in press).

22 For a classic discussion of personality disorder and self-harm, see Tantum and Whittaker (1993). Motz (2009a) is an excellent recent collection of articles about self-harm, particularly in relation to forensic contexts. See too Motz (2008) especially chapter 6, and the NICE Guidelines 2011 for longer-term management of self-harm. There are also an increasing number of patient self-reports available on-line and in medical textbooks and guidelines. Hawton, Saunders, and O'Connor (2012) is a comprehensive review of medical and social science studies of self-harm in adolescents. See Klonsky (2007) for a review of the evidence outlining the functions of self-harm.

23 Motz (2008, 2009a) contains moving self-reports and penetrating clinical understanding of self-harm. To give a mere flavour of some of these, consider the following self-report which describes self-harm as serving some, though not all, of the above functions: “I needed to kill something in me, this awful feeling like worms tunnelling along my nerves. So when I discovered the razor blade, cutting, if you'll believe me, was my gesture of hope. All the chaos, the sound and fury, the confusion and uncertainty and despair — all of it evaporated in an instant and I was for that minute grounded, coherent, whole. Here is the irreducible self. I drew the line in the sand, marked my body as mine, its flesh and its blood under my command” (quoted in Motz, 2009a, p. 47).

24 See references in footnote 11 and Pickard (in press) for further discussion.

25 Cf. Holton (2006), to which my understanding of the relationship between deliberation, choice, and action, is highly indebted.

26 For further discussion see Pickard (2013, in press).

27 For discussion of this form of argument in relation to addiction, see Pickard (2012, 2015).

28 Holton and Shute (2007) draw on philosophy and psychology to understand the nature of loss of control and link it to the defence of provocation. Although this defence was abolished and replaced with a loss of self-control defence under The Coroners and Justice Act 2009 Sections 54–6, which widens the emotions relevant to the defence, Holton and Shute's discussion of the philosophy and psychology is yet relevant. See too Horder (2005) for discussion of the role of fear alongside anger in loss of self-control.

29 For discussion see Buchanan and Brock (1989).

30 For discussion of the arguments and objections with respect to this position, see Craigie (2013). Note that it is important to acknowledge that diachronic change in desires and values can be normal and part of a developing and authentic self.

31 For discussion of the case see David et al. (2010) and Richardson (2013).

32 As described in *Novel on Yellow Paper* by Stevie Smith (1936).

33 Intriguingly, Szmukler suggests that the process of administering the test was itself therapeutic — offering the patients a quantity of time and quality of engagement that is rare, and which may indeed have enabled them to better think through their decisions to refuse treatment.

34 For discussion of this case, see Peay (2011a, 2011b).

35 Halpern (2012). For discussion see Craigie (2013) and Mackenzie and Watts (2011).

36 For the classic discussion of these findings written for a general audience, see Damasio (1994).

37 For the classic study supporting this finding, see Bechara, Damasio, Tranel, and Damasio (1997); see also Kahneman (2011).

38 For an important discussion of this phenomenon replete with well-crafted and rich examples, see Arpaly (2003), especially chapter 2. See too Jones (2004).

39 Cf. Charland (1998, 2011), Halpern (2012), and Mackenzie and Watts (2011).

40 For discussion of how treatment for personality disorder requires finding hope in face of this evidence and how narrative can be a useful clinical tool in doing so, see Pickard (2014).

41 Note that an argument with a similar conclusion can be made with respect to our obligations towards people with PD whose violence is other-directed. Given the backgrounds of extreme psycho-social and economic adversity typical of those with PD, our right to hold such offenders to account and punish may be predicated on a commitment to provision of adequate therapeutic and social, educational, and work opportunities that aim towards rehabilitation and reintegration – a very far cry from the present state of the criminal justice system. See Lacey and Pickard (2013, 2015).

42 For discussion of the nature of effective therapy for PD see NIMHE (2003), NICE (2004, 2011), Pearce and Pickard (2010), Pickard (2013, 2015), and Wood et al. (2014). For an ex-service user perspective, see Ward (2013) for a commentary on Pickard (2013). It is interesting to note the resonance between the clinical skills needed to support decision-making for those with PD, and the obligations on the state to support and enable decision-making of vulnerable people as articulated in the Convention on the Rights of Persons with Disabilities (CRPD). Cf. too *The Review of the Mental Health Act* Section 7.24 (Richardson et al., 1999, p. 95). For an extremely helpful discussion of threats and offers in mental health care, see Dunn et al. (2012).

43 See fn 41.
